# Antibiotic Action, Drug Delivery, Biodegradability, and Wound Regeneration Characteristics of Surgical Sutures and Cutting-Edge Surgical Suture Manufacturing Technologies

**DOI:** 10.3390/jfb16040135

**Published:** 2025-04-08

**Authors:** Hye-Ree Han

**Affiliations:** Department of Beauty Art Care, Dongguk University, Seoul 04620, Republic of Korea; luckyherry@hanmail.net; Tel.: +82-10-9130-8087

**Keywords:** surgical sutures, biodegradable, antibacterial, drug release, electrospinning, 3D printing

## Abstract

(1) Background: With the emergence of various super bacteria, interest in antibacterial properties, drug delivery, and wound regeneration is increasing in the field of surgical materials. There are many studies on surgical sutures, but not many recent ones that have studied structurally subdivided functions. Accordingly, various studies on surgical sutures were classified based on the main functions that are considered important, and studies were conducted by categorizing the latest production technology into 3D printing and electrospinning. (2) Methods: Data from the literature (n = 1077) were collected from databases such as PubMed, Harvard.edu, MDPI, Google Scholar, Web of Science, ACS, Nature, and IOP Publishing. The selected 103 papers were divided into two main groups: cutting-edge characteristics of surgical sutures and the latest technologies for manufacturing surgical sutures. (3) Results: Cutting-edge characteristics of surgical sutures were divided into four major categories: antibacterial, drug delivery, biodegradability, and wound regeneration, and examined in depth. In addition, the final technologies for manufacturing surgical sutures were divided into electrospinning and 3D printing. (4) Conclusions: The results of this study can contribute to the development of multifunctional surgical sutures that promote wound regeneration through antibacterial properties, drug elution, and biodegradability.

## 1. Introduction

With the emergence of new viruses, human society is facing a serious threat from the increased resistance of microorganisms to existing antibiotics. According to the World Health Organization (WHO), scientists around the world have argued that general infections and minor injuries can have serious consequences for human health if antibiotic resistance is not addressed [1].

The emergence of new super bacteria has further enhanced the importance of imparting antibacterial properties to surgical sutures. In addition, for patients at high risk of infection or with weak immune systems, surgical suture materials that can minimize infection should be chosen. Surgical sutures are the leading cause of in-hospital infections through surgical wound contamination, and their rates are increasing worldwide. An efficient way to prevent this is to modify the sutures’ surface to prevent bacteria from adhering [2].

**Figure 1:**
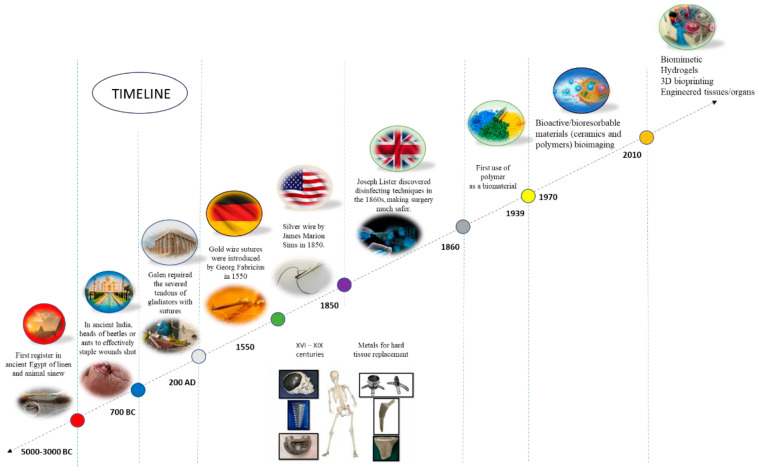
Schematic representation of the timeline indicating some of the main biomaterials data and the respective discoveries. Reprinted from ref. [3].

Figure 1 shows a schematic representation of the main biomaterials data and the timeline representing each discovery.

Antimicrobial sutures reduce the risk of infection at the surgical site through a coating of antimicrobial substances on general sutures.

On the other hand, studies on surgical suture materials have explored poly[(aminoethyl methacrylate)-co-(butyl methacrylate)] (PAMBM), catgut, and polyglycolic acid [PGA] sutures, and expanded polytetrafluoroethylene, silk sutures, collagen, chitosan, polyuronide, and polylactic acid [4,5]. In addition, many studies have been conducted in connection with antibiotic coating, Bio-inspired helical hollow bacterial cellulose fiber, oral surgery, bioelectric surgical suture, natural cellulose fiber, and site infection [6,7,8,9,10,11,12,13].

These materials exhibit human suitability, and it is important not to induce side effects such as human inflammatory reactions. External substances left in the human body are one of the most representative triggers of infection at the surgical site. In addition, for plastic surgery and cosmetic surgery, it is important to ensure natural results by reducing skin irritation and minimizing scars. Accordingly, there is an increasing demand for biodegradable sutures that naturally decompose in the body. For these reasons, biodegradability has also gained research attention.

A new trend in surgical materials is focused on natural resource materials with high biocompatibility, non-toxic properties, and specific properties aimed at the target site. In the past, polymer systems were used for simple sutures, but now their role is even more important because they need to heal surgical lesions or wounds, activate cell proliferation to refill damaged surfaces, protect damaged tissues and wounds from bacterial contamination, suppress infections, stop bleeding, and reabsorb moisture from wounds [14].

In addition, the importance of the drug delivery system for continuous and chronic wound treatment is increasing. Drug delivery is being studied with a focus on reducing wound areas, promoting cell migration, and maintaining continuous drug release, and research is being actively conducted to positively guide human recovery.

Although most sutures can provide sufficient mechanical support, their effects on clinical treatment are not satisfactory because they do not actively participate in wound healing. Therefore, it is very important to utilize biologically active sutures to promote wound healing. It has been reported that, after a wound occurs, a lateral wound electric field (EF) is formed from the surrounding intact tissue toward the center of the wound. Effective transmission of electrical signals generated by the wound EF can accelerate wound healing by regulating cellular behavior, including adhesion, proliferation, migration, and differentiation [15].

The purpose of this study is to examine, in depth, the main properties of surgical sutures that have recently been in the spotlight, including antibacterial properties, drug delivery systems, biodegradability, and wound regeneration. The application of electrospinning technology and 3D printing technology to the manufacture of surgical sutures is then considered. Next, based on the contents of this study, we tried to analyze the methods applied to multifunctional nanosurgical sutures, multifunctional mass production of 3D printing systems, and hybrid material manufacturing based on their use.

There are a lot of data on surgical sutures, but not many studies have structurally subdivided and explored the functions that have gained importance recently. Therefore, various studies on surgical sutures were classified based on the main functions (antibacterial, biodegradable, drug delivery, and wound regeneration), and the cases, strengths, and weaknesses of each were considered in depth. In addition, the latest technologies used for the production of surgical sutures were divided into 3D printing and electrospinning according to the manufacturing method, and a literature review was conducted.

## 2. Materials and Methods

**Figure 2:**
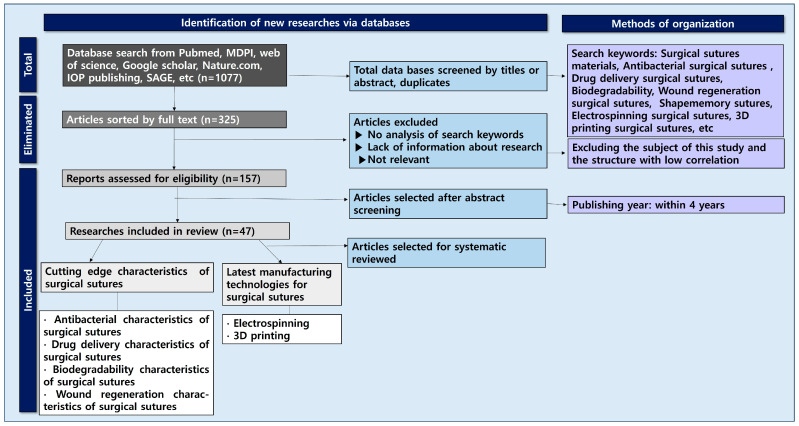
Flowchart of surgical sutures related to antibacterial, drug delivery, biodegradable, and wound regeneration characteristics and their production technologies.

In this study, an in-depth systematic review was conducted by classifying the main highlighted characteristics of surgical suture materials (Figure 2). Research data (n = 1077) were collected from databases such as PubMed, Harvard.edu, MDPI, Google Scholar, Web of Science, ACS, Nature, and IOP Publishing. Most of the documents used were papers published in the last 4 years (2021–2025). Keywords for the literature survey included ‘surgical suture drug delivery, surgical suture antibacterial coating, surgical suture material, surgical suture plant extract, surgical suture 3d printing, surgical suture electrospinning, biodegradability of surgical sutures, wound regeneration’. The databases containing the included studies were evaluated for further eligible publications. A total of 103 selected papers were divided into two major groups: cutting-edge characters of surgical sutures and the latest technologies for manufacturing surgical sutures.

Cutting-edge characteristics of surgical sutures were largely divided into four categories—antibacterial, drug delivery, biodegradability, and wound regeneration—and reviewed in depth.

In addition, the latest technologies for manufacturing surgical sutures were divided into electrospinning and 3D printing. In conclusion, this study aimed to provide a systematic review of the cornerstone applications of cutting-edge surgical suture material design.

## 3. Results and Discussion

### 3.1. Cutting-Edge Characteristics of Surgical Sutures

The main characteristics that have recently gained attention in surgical suture research include ‘Antibacterial, Drug delivery, Biodegradability, Wound regeneration’. In this section, these were divided into four topics and explored in depth.

#### 3.1.1. Antibacterial Characteristics of Surgical Sutures

Surgical sutures are used in almost all operations, but due to their lack of physiological signal responsiveness and their susceptibility to surgical site infection (SSI), their clinical treatment effectiveness is less satisfactory [15].

**Figure 3:**
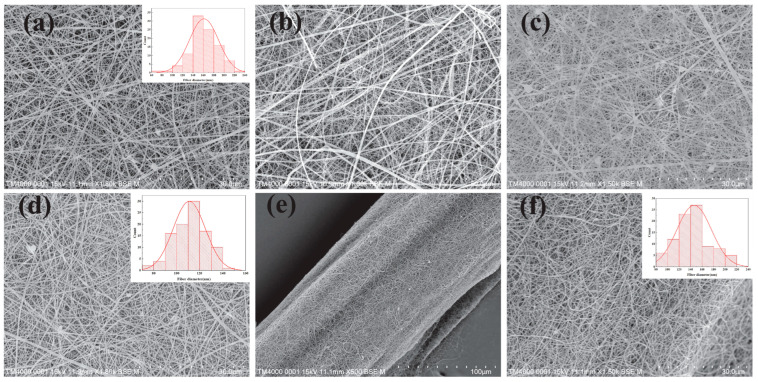
(**a**) SEM images of HPC/PVP electrospun nanofiber membrane; (**b**) SEM images of HPC/PVP-0.06% electrospun nanofiber membrane; (**c**) SEM images of HPC/PVP-0.07% electrospun nanofiber membrane; (**d**) SEM images of HPC/PVP-0.08% electrospun nanofiber membrane; (**e**,**f**) HPC/PVP/Zn electrospun nanofiber membrane and HPC/PVP/Zn electrospun wrapped yarns. Reprinted from ref. [16].

**Figure 4:**
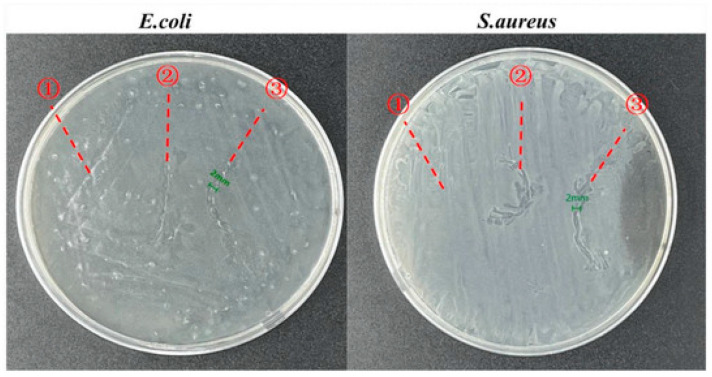
Zone of inhibitions for the in vitro antimicrobial activities of ① pure, ② HPC/PVP-coated, and ③ HPC/PVP-0.08% surgical sutures. Reprinted from ref. [16].

Figure 3 and Figure 4 show images of antimicrobial surgical sutures.

Therefore, studies on surgical sutures and antibacterial agents have been conducted in various fields, exploring chitosan, nanosilver particles, sodium carboxymethyl cellulose/chitosan/chlorhexidine, chitosan–gelatin/tannic acid/polypyrrole composite coating, utilization of supercritical carbon dioxide, curcumin@ ZIF-8, nylon monofilament, etc. [1,2,15,16,17,18,19,20,21,22,23,24,25,26,27,28,29,30,31,32,33,34,35,36,37,38,39,40,41,42,43]. Table 1 presents the articles on the antibacterial characteristics of surgical sutures, with their bibliographic sources.

James et al. argued that surgical sutures are the main cause of in-hospital infections through surgical wound contamination, with increasing rates worldwide. Among the methods for preventing infection is modifying the surface of the joint to prevent bacteria from adhering. In this study, antibacterial properties were obtained by coating a biopolymer polycaprolactone material containing nanosilver on the surface of the sutures. Biocompatible polyethylene glycol was selected as a solvent for dispersing nanosilver particles to improve the mechanical properties of the sutures [2].

Zhang et al. fabricated a step-by-step controlled antimicrobial suture (SZC) by coating the surface with Cur@ZIF-8(curcumin@zeolitic imidazolate framework-8). Taking advantage of the pH-responsive behavior of ZIF-8, Zn^2+^, and Cur(curcumin), it can be quickly released from acidic environments, increasing the efficiency of killing bacteria and preventing surgical site infections in a timely manner. The release of Zn^2+^ and Cur is reduced in a normal pH environment, protecting the cell/tissue from damage caused by excessive release [17].

Zhang et al. also argued that the current antimicrobial treatment method of silk sutures has the disadvantages of short antimicrobial effect, slow release, strong toxicity, and vulnerability to drug resistance, and these can only be addressed with surface modification. Recycled regenerated silk fibrin (RRSF) was extracted from waste silk resources to create an RRSF solution. Internal combination with organic titanium dioxide (TiO_2_) nanoparticles was carried out to produce an antimicrobial RRSF-based surgical suture. Surgical sutures with 1.25 wt% TiO_2_ obtained a knot strength of 2.40 N (diameter 143 μm) and achieved a sustainable antimicrobial effect of 93.58% [18].

Wang et al. prepared silk sutures with a braided core–shell structure from rubberized silk filaments for continuous double-drug delivery to prevent surgical site infections (SSIs), coating them with a silk fibrin (SF) layer loaded with berberine (BB) and artemisinin (ART). In an in vivo evaluation using Sprague Dawley (SD) mice, the coating was shown to reduce the expression of inflammatory cytokines interleukin-10 (IL-10) and tumor necrosis factor-α (TNF-α), thereby shortening the duration of inflammation and promoting angiogenesis [19].

Pajnik et al. were able to manufacture effective surgical sutures against *E. coli* and *Staphylococcus aureus* through the SSI process. All test samples showed antibacterial activity against *E. coli* and *S. aureus*, and this can be applied in the prevention of infection at the surgical site [1].

Altun et al. used a new manufacturing process based on pressure rotation for the first time to produce fiber surgical sutures by physically mixing antimicrobial triclosan (Tri) agents with poly(lactic-co-glycolic acid) (PLGA) and polyethylene oxide (PEO) polymers. All samples produced (5–40 TPP) showed increased inhibitory activity against *E. faecalis* and *E. coli* strains, depending on the Tri concentration loaded on fiber sutures. As expected, 40 TPP samples loaded with higher amounts of Tri exhibited higher antimicrobial activity compared with Vicryl Plus in all strains tested [20].

Zhang et al. made functional sutures by decorating the surface with a CS-GE/TA/PPy composite coating, which showed excellent antibacterial properties against *E. coli* and *Staphylococcus aureus*. Electroactive and antibacterial sutures greatly improved tissue regeneration [15].

As mentioned above, in order to impart antimicrobial properties to the surgical suture, the synthetic suture surface can be modified, or a nanosilver particle coating, coated with Cur@ZIF-8(curcumin@zeolitic imidazolate framework-8), recycled generated silk fibrin and TiO_2_, and mixed with PLGA and PEO, can be used to produce an antimicrobial surgical suture. It has been reported that these methods show excellent antimicrobial activity against bacteria such as *Staphylococcus aureus* or *E. coli* and can positively affect tissue regeneration.

#### 3.1.2. Drug Delivery Characteristics of Surgical Sutures

Deng et al. argued that sutures are the most popular surgical implants in the global surgical equipment market [44].

Studies on surgical sutures and drug delivery include research on multifunctional electronic sutures, anti-inflammatory silk sutures, chitosan/keratin/PCL/PEG drug elution sutures, scalable silk–fibrin-based berberine loading systems, hybrid hydrogel actuator, biopolymer–nanotube nerve guidance conduit drug delivery for peripheral nerve regeneration, etc. [14,19,24,44,45,46,47,48,49,50,51,52,53,54,55,56,57,58].

Table 2 presents the classified articles on drug delivery characteristics of surgical sutures, together with their bibliographic sources.

Deng et al. argued that natural polymers such as collagen, silk, nylon, and cotton, as well as synthetic polymers such as polycaprolactone, poly(lactic acid-co-glycolic acid), poly(p-dioxanone), etc., contribute to a solid foundation for drug elution suture engineering. In addition, it is possible to effectively address wound healing requirements by controlling drug elution profiles through manufacturing processes and the use of polymer materials [44].

Lee et al. argued that monitoring wound integrity and simultaneously promoting tissue regeneration remain challenging in the field of surgical sutures. To address this issue, they developed a drug-releasing electron sutures system (DRESS) to monitor suture integrity in real time and trigger drug release to improve tissue regeneration. The DRESS was fabricated using a single fiber of the core–shell structure consisting of a flexible conductive fiber core and a thermoreactive polymer shell containing the drugs. A layer of thermoreactive shell composed of flexible poly(vinyl alcohol) (PVA) grafted onto poly(*N*-isopropylacrylamide) (PNIPAm) facilitates drug release on demand through Joule heating. The results of an in vitro scratch assay showed a 66% reduction in wound area upon heat activation after 48 h, which demonstrated the efficacy of DRESS in stimulating response treatment by promoting cell migration [45].

Deng et al. fabricated diclophenac potassium load sutures based on PEG/PCL/chitosan/keratin mixtures using hot melt extrusion techniques. Optimal formulations of tensile strength were obtained when PCL/PEG/chitosan/keratin was combined at a ratio of 80/19/1 *w*/*w*, and rapid and continuous drug release rates were achieved with PEG/PCL/chitosan/keratin mixtures in various combinations. The complex of diclophenac potassium 30 wt% and PCL/PEG/chitosan/keratin exhibited high cell viability and wound healing rates in in vitro cytotoxicity tests [46].

Mendez et al. studied soft robotic drug delivery. Micro-CT imaging of the hybrid hydrogel actuator (HHA) prototype demonstrates robust and flexible adhesion to tissues during dynamic operation. The device enables coordinated mechanically reactive drug delivery directly to the target site and presents an innovative approach that integrates precisely controlled drug delivery with mechanical stimulation for enhanced topical treatment interventions [47].

Parikh et al. developed nanostructured multi-filament sutures capable of loading high-level low-molecular drugs while maintaining the high breaking strength required by the U.S.P. standard. The nanofiber-based sutures were shown to maintain strength even with a wide range of medications, deliver antibiotics to the eyes of mice for 30 days, and prevent ocular infections in a mouse model of bacterial keratitis. The multi-filament nanofiber sutures demonstrated biocompatibility and prevention of ophthalmic infections after multiple inoculations with *Staphylococcus aureus* for 1 week [48].

Manoukian et al. explained that chitosan-based nerve guidance conditions (NGCs) confirmed the continued release of 4-aminopyridine (4AP), a small-molecular drug that promotes neuroconductivity and neurotrophic factor release through aligned microchannel porosity and halosite nanotubes [49].

Bibire et al. recently explained that further modification of biomolecules using advanced technology can improve functions such as drug release capacity, wet adhesion performance, antibacterial activity, cell viability, and mechanical properties [14].

As mentioned above, drug elution profile control through polymer materials, weakly released electronic sutures systems, PEG/PCL/chitosan/keratin mixtures, micro-CT imaging of hybrid hydrogel actuator (HHA) prototypes, biopolymer modification, etc., can be used to produce drug delivery surges. These methods tended to have positive effects on human recovery, such as drug elution, wound site reduction, cell migration promotion, cell survival and wound healing rates, and continuous drug release.

#### 3.1.3. Biodegradability Characteristics of Surgical Sutures

Long-term use of non-degradable fiber implants can release some debris due to wear, which can be detrimental to surrounding cells and tissues, and can cause cell malformation, cell death, and even carcinogenesis [59].

Recent advances in biomedical engineering have highlighted the important role of biodegradable materials in solving challenges related to tissue regeneration therapy. The current spectrum of biodegradable materials includes ceramics, polymers, metals, and composites, each providing distinct benefits for the replacement or repair of damaged human tissues. Despite their utility, these biomaterials are not without limitations, and problems such as suboptimal tissue integration, potential cytotoxicity, and mechanical discrepancy (stress shielding) have emerged as serious concerns [60].

Studies on surgical sutures and biodegradability include research on oral surges, knot strength, orthopedic application, smart materials, self healing, silver nanowire, albumin composites, mechanical properties, absorbable sutures, PLA-based biopolymers, poly(lactic acid)/poly(ethylene glycol) blends, etc. [2,5,60,61,62,63,64,65,66,67,68,69,70,71,72,73].

Table 3 presents classified articles on the biodegradability characteristics of surgical sutures with bibliographic sources.

Absorbable sutures can be easily or completely absorbed into the body, and it is important to avoid postoperative wound infections and reduce patient pain. Absorbable sutures have been demonstrated to degrade by losing 50% of the tensile strength in tissues within 60 days. Among them, natural absorbent sutures are degraded by proteolysis, and synthetic absorbent sutures are degraded by hydrolysis. Absorbable suture materials are divided into natural absorbent suture materials and synthetic absorbent suture materials. Currently, commonly used absorbent suture materials include sheep intestines, poly-p-dioxanone (PDO), polyurethane (PU), polylactic acid (PLA), cellulose, polyglycolic acid (PGA), polycaprolactone (PCL), and poly(lactic-co-glycolic acid) (PLGA) [74].

Li et al. explained that compared to conventional non-degradable suture materials, absorbent materials exhibit unique properties that can be degraded in vivo without the need to be removed later, protecting patients from secondary trauma [65].

Antoniac et al. explained that under physiological conditions (pH = 7.4), irradiated surgical sutures with glycolide components degrade more rapidly, whereas surgical sutures made with polydioxanone do not. In addition, increasing pH leads to a higher degree of degradation and lower tensile strength, making it more likely that a wound will occur [61].

Nouri et al. found that biodegradable metals have high fixed strength and a low modulus of elasticity close to the bone, which facilitates bone union and allows the design of thinner and less bulky implants. Meanwhile, Mg has properties such as excellent biocompatibility, mechanical strength, and low modulus of elasticity similar to human bones. There are several competing approaches to slow down the decomposition/corrosion rate of Mg-based biomaterials, including coating, alloying, and surface treatment. Furthermore, Mg with abnormally high purity (>99.99%) has been demonstrated to degrade more slowly than other alloy systems. Using Mg and its alloys reduces MRI interference [62].

Naser et al. initiated the development of protein-based composites to alleviate shortcomings such as cytotoxicity, demonstrating improved biodegradability and biocompatibility; specifically, this study focused on the elaboration and characterization of innovative sutures fabricated with human serum albumin via extrusion methodology. It also shows that the use of proteins from human serum albumin (PHSA) can be utilized in the development of novel biodegradable sutures that can be used in many medical applications [60].

Szabelski et al. developed biopolymer composites by extensively fabricating filament sutures through a hot melt extrusion process based on human serum albumin protein. They studied the potential enhancements provided by the mixing of protein-based composites and biodegradable organic compounds, such as improved biodegradability and biocompatibility [63].

Wu et al. coated polylactic acid (PLLA) and polycaprolactone (PCL) on polydioxanone (PDO) sutures through a single-step method and showed that it increased the decomposition time and mechanical strength of the sutures in vitro and in vivo [64].

Alhulaybi et al. focused on producing absorbent surgical sutures from a biocompatible polymer material called polylactic acid (PLA) with PLA–chitosan composite sutures using an extrusion method for negative changes, followed by stretching methods. The sutures manufactured in this study were demonstrated to degrade in physiological saline. After 15 days, the sutures lost 50% of their weight, and their pH decreased from 6.49 to 4.42 [66].

As mentioned above, there are many studies on biodegradable metals, cellulose, PCL, PLA, etc., in absorbent materials, pH, protein-based composites, human serum albumin, and Mg that examine the biodiversity of surgical sutures. In addition, biodegradable materials made from the materials examined above tended to be more advantageous for protecting the human body or reducing pain than non-degradable materials.

#### 3.1.4. Wound Regeneration Characteristics of Surgical Sutures

Factors influencing wound healing include infection, necrosis, nutrition, medication, moisture, and physiological changes in individuals (age, immune system, stress, obesity, diabetes, etc.). The wound-healing process begins when a wound is formed after mechanical or chemical damage and includes a series of chemical signaling and cellular mechanisms, leading to regeneration and/or repair. Interventions to accelerate wound healing are essential because interruption of the healing process can lead to complications [28,75,76,77,78].

Research on wound regeneration and tissue regeneration using sutures includes studies on bioactive glass/graphene oxide-coated surgical sutures, reactive oxygen species-scavenging sutures, hybrid suture coating, and electrospun drug-eluting nanofibers [28,75,76,77,78].

Table 4 presents the classified articles on the wound regeneration characteristics of surgical sutures, together with their bibliographic sources.

Kerim Emre Öksüz et al. developed a novel bioactive glass/graphene oxide-coated surgical suture for soft tissue regeneration. In this experimental work, they demonstrated that graphene oxide (GO)-doped melt-derived bioactive bioglass nanopowders (BGNs) were synthesized through a sol-gel process. Subsequently, new GO-doped and undoped BGNs were used to coat the surgical sutures with poly[glycolide-co-lactide] surgical sutures to confer bioactive, biocompatible, and accelerated wound healing properties on the sutures. As a result, it was shown that the formation of BGN and GO on the sutures’ surface is significantly improved, thereby improving fibroblast attachment, migration, and proliferation and accelerating wound healing by promoting the secretion of angiogenesis growth factor [78].

Jiafei Zhu et al. developed a new ROS erasing suture by coating surgical sutures with gallic acid (GA)-based nanoparticles (GAMPs) for wound healing. Wound healing is a complex biological process involving hemostasis, cell proliferation, and tissue remodeling that is closely related to the level of oxidative stress in the damaged skin. The ROS cleaning sutures developed in this study enhance wound sutures, reduce inflammatory responses, and reduce scar formation, and can serve as promising sutures for wound healing under various conditions [77].

Ying-Ge Chen et al. developed mild-conditioned aqueous solutions without the use of organic solvents and preserved the basic properties of suturing materials based on the pH-dependent reversible self-polymerization of tannic acid (TA) and the strong adhesion of poly (tannic acid) (PTA). In the early postoperative stages, the wound site is susceptible to aseptic and/or bacterial inflammation. The acid conditions produced induce the explosive release of antimicrobial TA, mainly from adsorbed TA monomers. In the early postoperative stages, the wound site is susceptible to aseptic and/or bacterial inflammation. The acid conditions produced induce the explosive release of antimicrobial TA, mainly from adsorbed TA monomers [28].

Hui Han et al. studied a multifunctional surgical suture with electroactivity aided by oligochitosan/gelatin–tannic acid to promote skin wound healing and control scar proliferation. The multifunctional suture, designated S@LC@CGTP, had desirable in vitro persistent drug release properties that enabled Cur to be released for 8 days due to the action of PLGA. It has been shown that S@LC@CGTP suture materials have great potential to promote optimal, almost scar-free healing of surgical incision sites [76].

Nakamwi Akombaetwa et al. explained that in addition to the mechanical support provided by sutures and traditional wound dressing, therapeutic agents play an important role in accelerating wound healing. Drugs known to improve wound healing rate and extent include antibacterial, anti-inflammatory, and proliferation promoters. The development of these agents as eluted nanofibers has the potential to produce wound dressing and sutures that provide mechanical support with the benefit of locally delivering the therapeutic agent to the wound site [75].

As mentioned above, bioactive glass/graphene oxide coating, gallic acid (GA)-based nanoparticle (GAMP) coating, poly (tannic acid) utilization, and oligochitosan/gelatin-tannic acid can be used to produce surgical sutures. These methods tend to promote wound regeneration by improving fibroblast attachment, movement, and proliferation, promoting the secretion of vascular growth factors and enhancing antibacterial properties.

### 3.2. Latest Manufacturing Technologies for Surgical Sutures

#### 3.2.1. Electrospinning

**Figure 5:**
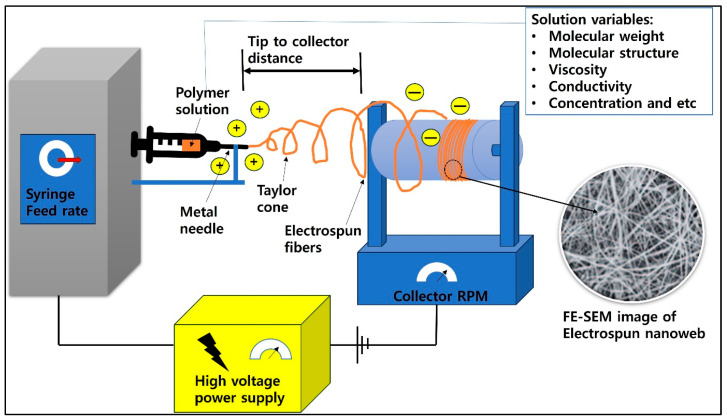
A schematic diagram of the electrospinning process.

Electrospinning technology can produce nano-thick fibers through high voltage and Van der Waals attraction. This nanotechnology is being actively studied in medical, fiber, and various engineering fields for reasons such as high surface area, possible core–sheath production, etc. A schematic of the electrospinning process is shown in Figure 5.

Articles on electrospinning surgery sealing include wound healing, inspection prevention, bioactive nano yarn, PLA, supercritical CO_2_ impregnation of ibuprofen and naproxen, poly-ε-caprolactone/propolis electrospun yarns, surface biofunctional bFGF-loaded electrospun suture, etc. [59,74,75,79,80,81,82,83,84,85,86,87,88,89,90,91,92,93,94,95,96,97,98,99].

Table 5 presents the classified articles on electrospinning technologies of surgical sutures, together with their bibliographic sources.

Xu et al. explained that, unlike single-fluid electrospinning techniques, double-fluid electrospinning spheres adopt double-layer composite superposition structures. There is a certain gap between the inner and outer layers so that the polymer solution flows smoothly. Finally, nanofibers of core–sheath structure are obtained. Using this manufacturing method, the performance can be altered by changing the position of the bioactive materials in the medical sutures’ nanofibers. For example, adding bioactive materials to the core solution slowly releases nanofibers’ control drugs and prolongs the sutures’ treatment time for the wound. When biologically active materials are added to the cover solution, the drug comes into direct contact with the wound, resulting in a wound treatment effect within a short period of time [74].

Teno et al. fabricated yarn based on flying entangled microfibers (1.95 ± 0.22 μm) in the field during the electrospinning process using a specially designed yarn collector. Electrospinning yarn sutures (300–500 μm in diameter) were made from poly(3-hydroxybutyrate-co-3-hydroxyvalerate) with different contents of 3HV units and contained ciprofloxacin hydrochloride (CPX) as an antibacterial activity pharmaceutical ingredient (API). All yarn sutures exhibited antibacterial properties during a 5-day time release against both Gram-positive and Gram-negative pathogenic bacteria [80].

Madheswaran et al. used electrospinning methods to fabricate chlorohexidine (CHX)-containing antimicrobial nanofiber covers around polyamide core threads. Cell conformity tests using 3T3-SA mouse fibroblasts and antimicrobial evaluations using *E. coli* and *Staphylococcus aureus* have suggested that CHX-containing PA6-based CNYs are biocompatible and have antimicrobial properties, suggesting that these threads can be used as functional and mechanically performing sutures [81].

Rivera et al. developed a poly(lactic acid) (PLA)-based suture using extrusion and electrospinning techniques followed by supercritical CO_2_ impregnation treatment. Polylactic acid (PLA) electrospinning fibers and films were impregnated with scCO2 with two nonsteroidal anti-inflammatory agents, ibuprofen and naphroxene, to obtain biodegradable suture materials with topical drug delivery capacity. A larger amount of the impregnated drug was obtained from the electrospinning PLA film, and 27% and 20% incorporation rates of naphroxene and ibuprofen, respectively, were obtained [82].

Wu et al. explained that the specific surface area increased significantly as the flow diameter reduced and that the ECM nanofiber yarns’ (NYs’) mimetic properties made electrospun nanofibers an ideal material for biomedical applications. More complex NY structures, including hollow NYs, double-layered coverspun NYs, and multilayerized coverspun Nys, were successfully produced. Numerous materials, including natural and synthetic polymers, as well as various functional additives, such as conductive materials, nanomaterials, and biologically active ingredients, have been successfully utilized to construct electrospun NYs with predetermined properties and functions [59].

Mohammedinooripoor et al. prepared yarn by applying an electrospinning composite mat consisting of poly-ε-caprolactone (PCL)/propolis ethanolic extrusion (PEE). In vitro cell culture studies on the biocompatibility of MG-63 and human skin fibroblasts in sutures and the antifungal and antifungal effects of sutures were also evaluated. Consequently, PEE-PCL sutures were shown to have elastic, mechanical, antifungal, and antifungal properties, as well as rapid wound-healing properties [83].

Li et al. fabricated a new degradable suture that controls the release of basic fibroblast growth factor (bFGF) for incisional wound healing. The suture (bFGF-DA@PCL) was fabricated by fixing bFGF to the surface of the electrospun polycaprolactone (PCL) suture with the help of dopamine. In vitro data demonstrated that the suture can effectively extend bFGF release by up to 10 days and improve cell adhesion and proliferation. Further in vivo results showed that the suture can increase wound healing and induce complete wound closure within 13 days. In addition, the suture was found to accelerate the mechanical recovery of the skin by restoring maximum intensity in healthy skin by 87.1% [84].

As mentioned above, it has been reported that electrospinning technology uses various substances to change the performance by changing the position of physiologically active substances or to show efficiency in imparting functions such as containing antibacterial active substances and excellent biodegradability. Electrospinning technology is expected to exert an excellent influence on the development of human-friendly surgical suture technology due to its thinness and high specific surface area.

#### 3.2.2. Three-Dimensional Printing

**Figure 6:**
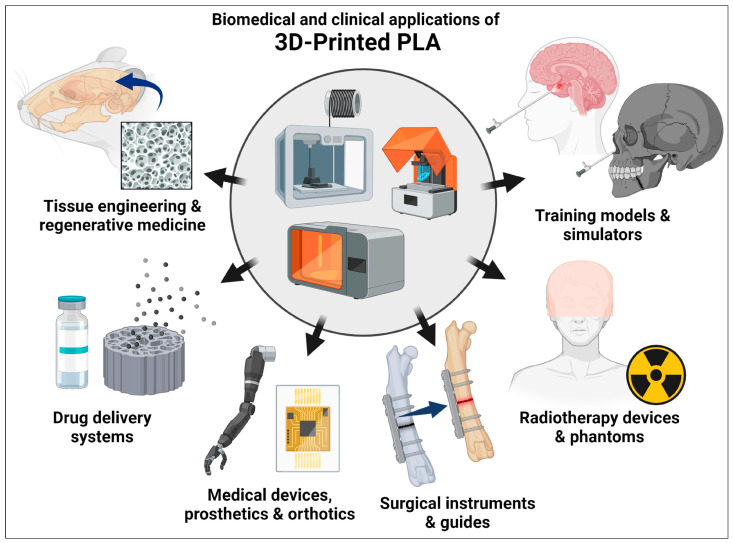
The schematic diagram related to 3D printing. Reprinted from ref. [100].

Three-dimensional printing technology has the ability to design products with computer programs without producing wastewater. The schematic diagram for 3D printing is shown in Figure 6.

Studies on 3D printing have explored biomimicry, bending behavior, suture fiber enhancement of gelatin scaffold, osteoporosis, artificial blood vessels, multi-material polymer composites, synthetic biomolecules, and elastic hydrogel conditions [101,102,103,104,105,106,107,108,109,110,111,112,113].

Table 6 presents the classified articles on 3D printing technologies for surgical sutures, together with their bibliographic sources.

Wickramasinghe et al. fabricated specimens through fused deposition modeling (FDM) using polylactic acid (PLA). They studied 3D-printed structures inspired by exoskeleton sutures of diabolic ironclad beetles. The sutures had the shape of an ellipse that meshes with each other, and the size was controlled by varying the lengths of the small and large radii of the ellipse, while the ratio between the radii was maintained at 1:1.8 [101].

Wickramasinghe et al. designed and 3D-printed specimens of diabolic ironclad beetle using polylactic acid (PLA) and studied the flexural behavior of the suture structure. The S3 design can be helpful when high energy absorption is required, and the S1 design can be very effective when higher load support is required. The results of this study argued that the bio-inspired suture structure can be further optimized to improve the performance [102].

Nash et al. fabricated a single-wrap shear specimen of a bio-inspired suture interface through a layer-by-layer polymer injection lamination fabrication technique (Stratasys connex) for multi-material printing. As a result, they showed that by increasing the waveform of the suture layer, the effective shear stiffness of the junction can be significantly increased. For certain designs in this paper, wavy sutures can reach an effective shear stiffness 10–20 times greater than that of flat sutures [103].

Choi et al. improved the printability, mechanical strength, and dimensional stability of 3D-printed scaffolds by adding biodegradable suture fibers to gelatin biomaterial ink. The suture fibers increased the mechanical strength of the 3D-printed scaffold by up to sixfold, and the rate of deterioration could be controlled by the suture fiber content. In an in vitro cell study, DNA analysis revealed that the human skin fibroblast proliferation rate of 3D-printed skeletons containing 0.5% suture fibers was 10% higher than that of 3D-printed skeletons without suture fibers after 14 days of incubation [104].

Zhou et al. argued that damaged blood vessels should be replaced when cardiovascular disease is severe. They also developed a new and simple 3D mold fabrication technology that can be used to prepare 3D molds of various sizes. They prepared a double cross-linking (ionic cross-linking between Ca^2+^ and sodium alginate) and free radio polymerization of acrylamide (using *N*,*N*′-methylenebisacrylamide as a cross-linking agent), resulting in polyacrylamide/*N*,*N*′-methylenebisacrylamide/sodium alginate (PMSA) hydrogel tube using this method. The manufactured PMSA hydrogel tube has excellent mechanical properties and biocompatibility and can be adjusted to within 6 mm in diameter, so it can be used as a small-diameter vascular graft [105].

Xu et al. adjusted the composition of the hydrogel matrix and the immersion time of the ionic solution to accurately match the different biological soft tissues to complete a dual-network (DN) hydrogel that mimics tissue with custom stiffness. Combined with advanced three-dimensional (3D) printing fabrication technology, they perfected a variety of performance-adjustable biological hydrogel long-term models with structural complexity and fidelity, including kidney, liver, pancreas, and vascular tissue [106].

Altuntas et al. explained that the suture interface formed between the two different polymer phases of PLA (hard) and TPU (soft) was designed and fabricated by a molten filament fabrication technique. As a result, it was argued that lamination fabrication could provide a guideline for producing a multi-material polymer composite material with a stronger and more robust interface [107].

As mentioned above, many studies on mechanical strength and dimensional stability have been reported in 3D printing using lamination manufacturing technology and biomaterials. Three-dimensional printing technology is expected to have a positive effect on the development of human-friendly surgical suture technology due to the convenience of structural design.

## 4. Conclusions

Antibiotic properties, drug delivery, biodegradability, and wound regeneration, which are the main characteristics of surgical sutures, were examined in depth in this study. In addition, the final technologies for manufacturing surgical sutures were divided into electrospinning and 3D printing.

- Current status

Research on surgical sutures and antibacterial properties proposes methods such as modifying the yarn surface or coating with nanosilver particles, coating with Cur@ZIF-8, utilizing recycled silk–fibrin and TiO_2_, and coating the chitosan–gelatin/tannic acid/polypyrrole composite. These methods have been reported to produce excellent antibacterial activity against super bacteria (staphylococcus aureus, *E. coli*, etc.). 

Studies on surgical sutures and drug delivery explore the use of anti-inflammatory silk sutures, chitosan/keratin/PCL/PEG drug elution sutures, scalable silk–fibrin-based berberine loading system, and hybrid hydrogel actuators. Some of these methods have been reported to positively affect human recovery, such as drug elution, wound site reduction, cell migration promotion, high cell survival and wound healing rates, and continuous drug release.

Studies on the biodegradability of surgical sutures explore orthopedic applications, knot strength, smart materials, self healing, silver nanowire, albumin composites, mechanical properties, absorbable sutures, PLA-based biopolymers, poly(lactic acid)/poly (ethylene glycol) blends, etc. In addition, it is reported that biodegradable materials are more advantageous in protecting the human body and reducing pain than non-degradable materials.

Reactive oxygen species-scavenging sutures, hybrid suturing coating, and electrospun drug-eluting nanofibers are explored in the studies on wound regeneration and tissue regeneration using sutures. Using these methods, fibroblast attachment, migration, and proliferation are improved, the secretion of vascular growth factors is promoted, and wound healing is reported.

Electrospinning technology has been reported in many cases to change performance by changing the position of physiologically active substances using various substances or to show efficiency in imparting functions such as antibacterial-active substances and excellent biodegradability. Electrospinning technology is expected to exert an excellent influence on the development of human-friendly surgical suture technology due to its thinness and high specific surface area.

Research on 3D printing includes studies on biomimicry, bending behavior, artificial blood vessels, multi-material polymer composites, and elastic hydrogel conditions. As mentioned above, many studies on mechanical strength and dimensional stability using lamination manufacturing technology and biomaterials have been reported for 3D printing. Three-dimensional printing technology is expected to have a positive effect on the development of human-friendly surgical suture technology due to the convenience of structural design.

- The main obstacles to be addressed

There are many studies on antibiotic action, drug delivery, biodegradability, and wound regeneration of surgical sutures; however, side effects include toxicity.

In Korea, it is a violation of the Medical Device Management Act for a doctor to modify or modulate a general medical thread for the purpose of removing facial wrinkles. After the procedure, the skin wrinkle removal procedure usually involves swelling and pain, and since the skin wrinkle removal thread is inserted directly into the body, it is necessary to ensure its stability. If the skin wrinkle removal thread is arbitrarily manufactured using a general surgical thread, the protrusion spacing may not be uniform and may be partially broken, and the production process may be contaminated or deteriorated; thus, the possibility of side effects cannot be excluded. Therefore, the Korean court would convict the prosecution in this case, considering that the act of arbitrarily making protrusions in the general surgical thread and using it for skin wrinkle removal constitutes tampering with and modulation of medical devices. Due to these precedents, attention should be paid to the modification, modulation, and misuse of surgical sutures.

- Future perspectives

With the emergence of new viruses, research on antimicrobial properties, biodegradability for human suitability, and drug delivery systems for rapid healing are expected to be further promoted, along with mass production technology. In addition, the results of this study are considered to have a positive effect on the development of a multifunctional sur-chemical suture that promotes wound regeneration through antibacterial properties, drug elution, and biodegradability.

In this study, the main characteristics required for surgical sutures in the current society where new bacteria appear were considered from various angles in terms of antibacterial, biodegradable, drug delivery, and wound regeneration properties. In addition, electrospinning techniques that can produce nanofibers and 3D printing technologies that are output in a stacked form according to computer design were examined. In the present era of requiring multifunctional surgical sutures, the implications and practical significance of this study are expected to provide the foundation for the manufacture of hybrid surgical sutures. In addition, future research is expected to provide useful information for the surgical sutures industry if the functional materials mentioned in this study are fused to apply appropriate surgical sutures according to the wound site and type, age, race, and gender.

## Figures and Tables

**Table 1:** Articles on the classified antibacterial characteristics of surgical sutures, with bibliographic sources.

Authors	Title	Main Contents of Antibacterial Characteristics	Effect of the Research	Bibliographic Source
James, B et al.	Development of environmentally safe biodegradable, antibacterial surgical sutures using nanosilver particles.	Antibacterial surgical sutures, coating a biopolymer, nano silver particles, environmentally safe, biodegradable	Antimicrobial properties were obtained by coating a biopolymer polycaprolactone material with nanosilver on the suture surface. Biocompatible polyethylene glycol was selected as the nanosilver particle dispersion solvent to improve the mechanical properties of the suture.	Journal of Polymers and the Environment, 2021. 29(7): p. 2282–2288 [2].
Zhang, Q et al.	Stage-controlled antibacterial surgical sutures based on curcumin@ ZIF-8 functional coating for improved wound healing.	Antibacterial surgical sutures, *E. coli* and *S. aureus*, curcumin@ ZIF-8, SZC, wound healing, organic coatings	SZC exhibited excellent antimicrobial properties against *E. coli* and *S. aureus*. SZC has excellent mechanical properties and handling performance.	Progress in Organic Coatings, 2023. 184: p. 107829 [17].
Zhang, X et al.	Sustainable Antibacterial Surgical Suture Based on Recycled Silk Resource by an Internal Combination of Inorganic Nanomaterials.	Antibacterial surgical sutures, recycled silk resources, inorganic nanomaterials, wound healing.	The surgical suture with 1.25 wt% TiO_2_ had a 2.40 N knot strength (diameter 143 μm) and achieved a sustainable antibacterial effect of 93.58%. Surprisingly, this suture significantly reduced the inflammatory response and promoted wound healing.	ACS applied materials & interfaces, 2023. 15(25): p. 29971–29981 [18].
Wang, X et al.	Sustainable antibacterial and anti-inflammatory silk suture with surface modification of combined-therapy drugs for surgical site infection.	Antibacterial, anti-inflammatory, Sprague Dawley (SD) mice, silk suture, surgical site infection.	In vivo evaluation using Sprague Dawley (SD) mice showed that the coating reduced the expression of the inflammatory cytokines interleukin-10 (IL-10) and tumor necrosis factor-α (TNF-α), thus shortening the duration of inflammation and promoting angiogenesis. The results showed that these novel sutures exhibited stable structure, good biocompatibility, sustainable antimicrobial, and anti-inflammatory functions, and had surgical applicability.	ACS Applied Materials & Interfaces, 2022. 14(9): p. 11177–11191 [19].
Pajnik, J et al.	Utilization of supercritical carbon dioxide for development of antibacterial surgical sutures.	Antibacterial, *E. coli*, *Staphylococcus aureus*, supercritical carbon dioxide, antibacterial surgical sutures.	The SSI process allowed the manufacture of surgical sutures effective against *E. coli* and *Staphylococcus aureus*. Mild process conditions (35 °C, 10 MPa, 1–6 h) allowed up to 5.9% of thymol loading.	The Journal of Supercritical Fluids, 2022. 181: p. 105490 [1].
Altun, E et al.	Pressure-Spun Fibrous Surgical Sutures for Localized Antibacterial Delivery: Development, Characterization, and In Vitro Evaluation.	Pressure-spun fibrous surgical sutures, antibacterial delivery, vitro evaluation.	After 24 h of in vitro antimicrobial testing, sutures with 285 ± 12 μg/mg Tri loading exhibited antimicrobial activity against all tested bacterial strains.	ACS applied materials & interfaces, 2023. 15(39): p. 45561–45573 [20].
Zhang, Q et al.	Electroactive and antibacterial surgical sutures based on chitosan-gelatin/tannic acid/polypyrrole composite coating.	Antibacterial surgical sutures, *E. coli* and *Staphylococcus aureus*, CS-GE/TA/PPy, electroactive, chitosan–gelatin/tannic acid/polypyrrole	Functional sutures showed excellent antibacterial properties against *E. coli* and *Staphylococcus aureus*. Functional sutures are conductive even when knotted, ensuring stable electrical signal transmission.	Composites Part B: Engineering, 2021. 223: p. 109140 [15].

**Table 2:** Articles on classified drug delivery characteristics of surgical sutures with their bibliographic sources.

Authors	Title	Main Contents of Drug Delivery Characteristics	Effect of the Research	Bibliographic Source
Deng, X et al.	Engineering and polymeric composition of drug-eluting suture: a review.	Drug-eluting, polymeric composition, wound healing	Natural polymers such as lagen, silk, nylon, and cotton, and synthetic polymers such as polycaprolactone, poly(lactic-co-glycolic acid), and poly(p-deoxanone) provide a solid foundation for drug elution suture engineering. Manufacturing processes and polymer materials can effectively address wound healing requirements by controlling drug elution profiles.	Journal of Biomedical Materials Research Part A, 2021. 109(10): p. 2065–2081 [44].
Lee, Y et al.	A multifunctional electronic suture for continuous strain monitoring and on-demand drug release.	Drug release, multifunctional electronic suture, continuous strain monitoring	A thermoreactive shell layer consisting of flexible poly(vinyl alcohol) (PVA) grafted onto poly(*N*-isopropylacrylamide) (PNIPAm) facilitates on-demand drug release through Joule heating. In vitro scratch analysis demonstrated the efficacy of DRESS in stimulation response treatment by reducing the wound area by 66% upon heat activation after 48 h, facilitating cell migration.	Nanoscale, 2021. 13(43): p. 18112–18124 [45].
Deng, X et al.	Fabrication and characterisation of melt-extruded chitosan/keratin/PCL/PEG drug-eluting sutures designed for wound healing.	Drug-eluting, melt-extruded, chitosan/keratin/PCL/PEG, wound healing	A literature study comparing drug release in pure PCL and PCL/PEG blends found that the pure PCL matrix released only 4% of the drug over a 7-day period. In contrast, in the case of a polymer blend with a hydrophilic component of 30%, more than 80% of the drug was released within 72 h. In the current study, up to 20% of PEG was used, and the duration of rapid release of the drug was related to the time point at which the PEG began to decompose.	Materials Science and Engineering: C, 2021. 120: p. 111696 [46].
Mendez, K et al.	Mechanoresponsive Drug Release from a Flexible, Tissue-Adherent, Hybrid Hydrogel Actuator.	Drug release, hybrid hydrogel actuator, flexible, tissue-adherent	In this study, a new type of hybrid hydrogel actuator (HHA) was introduced to facilitate enhanced drug delivery. Multi-material soft actuators can induce controllable mechanoreactive release of charged drugs in the alginate/acrylamide hydrogel layer through temporal control.	Advanced Materials, 2024. 36(43): p. 2303301 [47].
Parikh, K.S et al.	Ultra-thin, high strength, antibiotic-eluting sutures for prevention of ophthalmic infection.	Antibiotic-eluting sutures, prevention of ophthalmic infection, ultra-thin, high strength.	Nanofiber-based sutures maintained strength even when loaded with a wide range of drugs, administered antibiotics for 30 days in the eyes of mice, and prevented eye infections in a bacterial keratitis mouse model. The nanofiber-based polycaprolactone sutures did not decrease in strength when loaded with 8% levofloxacin, whereas the monofilament sutures decreased in strength by more than 50%.	Bioengineering & Translational Medicine, 2021. 6(2): p. e10204 [48].
Manoukian, O.S et al.	Biopolymer-nanotube nerve guidance conduit drug delivery for peripheral nerve regeneration: In vivo structural and functional assessment.	Drug delivery, biopolymer-nanotube, in vivo structural peripheral nerve regeneration.	Continuous 4-aminopyridine release amplifies neurotrophic factor release by Schwann cells, promoting axon regeneration. The TGA derivative curve shows a drug loading of 7.69% by weight within the range of 5–10% established by successful drug loading of HNT, which has not been modified in the literature. NGC was able to provide a continuous release of 4-AP for 8 weeks.	Bioactive Materials, 2021. 6(9): p. 2881–2893 [49].
Bibire, T et al.	Biopolymers for surgical applications.	Drug delivery, biopolymer, excellent wet adhesion performance, antimicrobial activity, cell viability	Various combinations of PEG/PCL/chitosan/keratin mixtures achieved rapid and sustained drug release rates. PCL/PEG/chitosan–keratin complex with 30% of diclophenac potassium by weight showed high cell viability and wound healing rates during in vitro cytotoxicity testing.	Coatings, 2022. 12(2): p. 211 [14].

**Table 3:** Classified articles on the biodegradability characteristics of surgical sutures with their bibliographic sources.

Authors	Title	Main Contents of Biodegradability Characteristics	Effect of the Research	Bibliographic Source
Antoniac, I et al.	In vitro study on biodegradation of absorbable suture Materials used for surgical applications.	Biodegradation, in vitro study, absorbable suture materials, surgical applications	Depending on the pH value, it is observed that at pH = 7.4, the sample decomposes faster than under acidic conditions. In addition, important aspects were highlighted for samples P2 (lactic acid-co-glycolic acid) and P5 (poly(glycolide-co-ε-caprolactone).	Mater. Plast, 2021. 58: p. 130–139 [61].
Nouri, A et al.	Biodegradable metallic suture anchors: A review.	Biodegradable, metallic suture anchors, MG, implant	Since AM technology was only recently introduced in orthopedics, the issues related to its effectiveness in bone anchor manufacturing are still unresolved. Only a small number of natural polymers, including collagen, keratin, and chitosan, have been found as suitable biodegradable implant materials.	Smart Materials in Manufacturing, 2023. 1: p. 100005 [62].
Naser, M.A et al.	Biodegradable suture development-based albumin composites for tissue engineering applications.	Biodegradable suture, tissue engineering, albumin composites	Preliminary tensile tests described the mechanical profile of filament sutures (FSs), with tensile strengths ranging from 1.3 to 9.616 MPa and fracture elongation rates ranging from 11.5 to 146.64%. These results reveal the mechanical diversity of sutures, suggesting their applicability to a wide range of medical interventions.	Scientific Reports, 2024. 14(1): p. 7912 [60].
Szabelski, J et al.	Short-Term Hydrolytic Degradation of Mechanical Properties of Absorbable Surgical Sutures: A Comparative Study.	Hydrolytic degradation, absorbable surgical sutures, protein composites	SafilQuick+ and MonosynQuick sutures lost their strength within 9 to 12 days, as evidenced by statistically significant changes in tensile strength. Surgical sutures play an important role in wound sutures and facilitate tissue healing processes in various medical fields.	Journal of Functional Biomaterials, 2024. 15(9): p. 273 [63].
Wu, H et al.	Surface coating prolongs the degradation and maintains the mechanical strength of surgical suture in vivo.	Degradation, surgical sutures in vivo, surface coating, mechanical strength	Absorbable sutures undergo decomposition and absorption during tissue repair, whereas nonabsorbable sutures maintain tensile strength and resist absorption in combination with healing. In anterior cruciate ligament repair, which requires a long time to heal, nonabsorbable sutures outperformed absorbent sutures after 15 weeks of observation.	Colloids and Surfaces B: Biointerfaces, 2022. 209: p. 112214 [64].
Alhulaybi, Z.A et al.	Fabrication and Characterization of Poly(lactic acid)-Based Biopolymer for Surgical Sutures.	Biodegradable, PLA, surgical Sutures, biopolymer	According to decomposition studies, the sutures manufactured in this study were demonstrated to degrade in physiological saline. After 15 days, the sutures lost 50% of their weight, and their pH decreased from 6.49 to 4.42.	ChemEngineering, 2023. 7(5): p. 98 [66].

**Table 4:** Classified articles on the wound regeneration characteristics of surgical sutures and their bibliographic sources.

Authors	Title	Main Contents of Wound Regeneration Characteristics	Effect of the Research	Bibliographic Source
Öksüz, K.E., et al.	Novel Bioactive Glass/Graphene Oxide-Coated Surgical Sutures for Soft Tissue Regeneration	Soft tissue regeneration, bioactive glass/graphene oxide, surgical sutures	The highest cell activity was observed in the S + BGNs (95.5 ± 1.23%) and S + BGNs/GO (86.73 ± 2.83%) groups. However, there was a noticeable difference in cell activity in the S group (80.87 ± 1.93%), and consequently, it proved beneficial for cell growth because of the presence of a mixed polycrystalline hydroxyl–carbonate–apatite (HCA) layer on the suture surface.	ACS omega, 2023. 8(24): p. 21628–21641 [78].
Zhu, J et al.	Reactive Oxygen Species Scavenging Sutures for Enhanced Wound Sealing and Repair	Reactive oxygen species, scavenging sutures, wound sealing and repair	The obtained sutures coated with GANP can effectively promote wound closure by maintaining tension around the wound and reducing the ROS level. Specifically, the GANP coated on the sutures can effectively eliminate ROS, upregulate anti-inflammatory molecules, and polarize macrophages around the wound site by M2 phenotype due to the efficient antioxidant activity of GA, a type of low molecular weight difference polyphenol.	Small Structures, 2021. 2(7): p. 2100002 [77].
Chen, Y.-G et al.	Hybrid suture coating for dual-staged control over antibacterial actions to match well wound healing progression	Dual-staged control, wound healing progression, hybrid suture coating, antibacterial	Acidic conditions induce the explosive release of antimicrobial TA, mostly from adsorbed TA monomers. In the later stages, TA release is fully tuned to pH conditions, which depend on the degree of healing of the wound, enabling continuous antimicrobial prevention in a biologically controllable manner.	Materials Horizons, 2022. 9(11): p. 2824–2834 [28].
Han, H et al.	A multifunctional surgical suture with electroactivity assisted by oligochitosan/gelatin-tannic acid for promoting skin wound healing and controlling scar proliferation	Electroactivity assisted, oligochitosan/gelatin–tannic acid, skin wound healing, controlling scar proliferation	Through in vivo experiments, S@LC@CGTP was able to alleviate inflammation and promote scar-free wound healing following the suppression of invasive inflammatory cells, downregulation of TGF-β1 and type I collagen expression, and improved collagen arrangement. Cumulatively, we have shown that S@LC@CGTP suture materials have great potential in promoting optimal, almost scar-free healing of surgical incisions.	Carbohydrate Polymers, 2023. 320: p. 121236 [76].
Akombaetwa, N et al.	Applications of Electrospun Drug-Eluting Nanofibers in Wound Healing: Current and Future Perspectives	Wound healing, drug-eluting, electrospun nanofibers	Running sutures were inserted into the non-abdominal muscles of male Wistar mice at a concentration of 0.1 or 1 µg. VEGF/poly(l-lactide)(PLA) blend coatings showed increased biological activity and cell viability.	Polymers, 2022. 14(14): p. 2931 [75].

**Table 5:** Classified articles on the electrospinning technologies of surgical sutures with their bibliographic sources.

Authors	Title	Main Content	Bibliographic Source
Xu, L et al.	Electrospun medical sutures for wound healing: A review.	Electrospinning, wound healing, medical sutures	Polymers, 2022. 14(9): p. 1637 [74].
Teno, J et al.	Development of Ciprofloxacin-Loaded Electrospun Yarns of Application Interest as Antimicrobial Surgical Suture Materials.	Electrospun yarns, ciprofloxacin, antimicrobial, surgical suture materials	Pharmaceutics, 2024. 16(2): p. 220 [80].
Madheswaran, D et al.	Composite yarns with antibacterial nanofibrous sheaths produced by collectorless alternating-current electrospinning for suture applications.	Electrospinning, composite yarns, antibacterial nanofibrous sheaths, suture applications.	Journal of Applied Polymer Science, 2022. 139(13): p. 51851 [81].
Rivera, P et al.	Development of PLA suture materials by extrusion, electrospinning and supercritical CO_2_ impregnation of ibuprofen and naproxen.	Electrospinning, PLA suture materials, supercritical CO_2_ impregnation, ibuprofen, naproxen	The Journal of Supercritical Fluids, 2023. 194: p. 105854 [82].
Wu, S et al.	State-of-the-art review of advanced electrospun nanofiber yarn-based textiles for biomedical applications.	Electrospun nanofiber yarn, biomedical applications, double-layered coverspun NYs, multilayered coverspun	Applied Materials Today, 2022. 27: p. 101473 [59].
Mohamadinooripoor, R et al.	Poly-ε-Caprolactone/Propolis Electrospun Yarns as Suture.	Electrospun yarns, Poly-ε-Caprolactone, propolis, sutures	Fibers and Polymers, 2023. 24(8): p. 2641–2651 [83].
Li, Y et al.	Surface biofunctional bFGF-loaded electrospun suture accelerates incisional wound healing.	Electrospun suture, incisional wound healing, surface biofunctional bFGF	Materials & Design, 2023. 225: p. 111451 [84].

**Table 6:** Classified articles on 3D printing technologies for surgical sutures and their bibliographic sources.

Authors	Title	Main Content	Bibliographic Source
Wickramasinghe, S et al.	Analysing fracture properties of bio-inspired 3D printed suture structures.	3D printing, bio-inspired structure fracture properties	Thin-Walled Structures, 2022. 176: p. 109317 [101].
Wickramasinghe, S., T et al.	Flexural behavior of 3D printed bio-inspired interlocking suture structures.	3D printing, bio-inspired, interlocking suture structures, flexural behavior	Mater. Sci. Addit. Manuf, 2022. 1(9) [102].
Nash, R.J et al.	Experimental and numerical analysis of 3D printed suture joints under shearing load.	3D printing, suture joints, shearing load	Engineering Fracture Mechanics, 2021. 253: p. 107912 [103].
Choi, D.J et al.	Suture fiber reinforcement of a 3D printed gelatin scaffold for its potential application in soft tissue engineering.	3D printing, soft tissue engineering, suture fiber, gelatin scaffold	International journal of molecular sciences, 2021. 22(21): p. 11600 [104].
Zhou, L et al.	A 3D printing mold method for rapid fabrication of artificial blood vessels.	3D printing, artificial blood vessels, polyacrylamide/*N*,*N*′-methylenebisacrylamide/sodium alginate (PMSA) hydrogel tube	Colloids and Surfaces A: Physicochemical and Engineering Aspects, 2023. 662: p. 130952 [105].
Xu, X., et al. et al.	Multifunctional high-simulation 3D-printed hydrogel model manufacturing engineering for surgical training.	3D printing, hydrogel model, manufacturing engineering for surgical training	International Journal of Bioprinting, 2023. 9(5) [106].
Altuntas, U., D. Coker et al.	Creating tougher interfaces via suture morphology in 3D-printed multi-material polymer composites by fused filament fabrication.	3D printing, multi-material polymer composites, suture morphology, fused filament fabrication.	Additive Manufacturing, 2023. 61: p. 103359 [107].

## Data Availability

Not applicable.

## References

[1] Pajnik J., Milovanovic S., Stojanovic D., Dimitrijevic-Brankovic S., Jankovic-Častvan I., Uskokovic P. (2022). Utilization of supercritical carbon dioxide for development of antibacterial surgical sutures. J. Supercrit. Fluids.

[2] James B., Ramakrishnan R., Aprem A.S. (2021). Development of environmentally safe biodegradable, antibacterial surgical sutures using nanosilver particles. J. Polym. Environ..

[3] Ornaghi H.L., Monticeli F.M., Agnol L.D. (2023). A Review on Polymers for Biomedical Applications on Hard and Soft Tissues and Prosthetic Limbs. Polymers.

[4] Li Y., Kumar K.N., Dabkowski J.M., Corrigan M., Scott R.W., Nüsslein K., Tew G.N. (2012). New Bactericidal Surgical Suture Coating. Langmuir.

[5] Faris A., Khalid L., Hashim M., Yaghi S., Magde T., Bouresly W., Hamdoon Z., Uthman A.T., Marei H., Al-Rawi N. (2022). Characteristics of suture materials used in oral surgery: Systematic review. Int. Dent. J..

[6] Wang J., Liu Z., Qiu H., Wang C., Dong X., Du J., Li X., Yang X., Fang H., Ding Y. (2025). A robust bio-based polyurethane employed as surgical suture with help to promote skin wound healing. Biomater. Adv..

[7] Chung D., Bakmiwewa S., Suthananthan A., Idrees M. (2025). Suture material in pancreticojejunal anastomosis: A systematic review. ANZ J. Surg..

[8] Paladini F., Panico A., Masi A., Russo F., Sannino A., Pollini M. (2025). Silver-Treated Sutures for the Prevention of Biofilm-Associated Surgical Site Infections. Antibiotics.

[9] Rineksa G., Whulanza Y., Gozan M. (2025). Mechanical characteristics of thermoplastic sago starch-based biopolymer composite reinforced with microcrystalline cellulose (MCC) as a potential surgical suture material. Proceedings of the AIP Conference Proceedings.

[10] Das S., Ghosh D., Khatib M.N., Balaraman A.K., Roopashree R., Kaur M., Srivastava M., Barwal A., Prasad G.S., Rajput P. (2025). Bioelectric surgical sutures: Advancing wound healing through mechano-electrical stimulation. Int. J. Surg. Open.

[11] Makrygiannis I.H., Nikolaidis A.K., Tilaveridis I., Kouvelas A.D., Lykakis I.Ν., Venetis G. (2025). Coated sutures for use in oral surgery: A comprehensive review. Clin. Oral Investig..

[12] Zhou Y., Liu X., Yang M., Song G., Wang Y., Sun H., Yuan T., Rao J., Lü B., Yao C. (2025). Bio-inspired helical-hollow bacterial cellulose fiber for suture materials. Chem. Eng. J..

[13] Abdulagatov I.M., Khanaliev V.Y., Ragimov R.M., Maksumova A.M., Khamidov M.A., Abdullaeva N.M., Mollaeva N.R. (2025). Atomic-layer-deposition application for antibacterial coating of biomedical materials: Surgical sutures. Biomed. Mater..

[14] Bibire T., Yilmaz O., Ghiciuc C.M., Bibire N., Dănilă R. (2022). Biopolymers for surgical applications. Coatings.

[15] Zhang Q., Qiao Y., Zhu J., Li Y., Li C., Lin J., Li X., Han H., Mao J., Wang F. (2021). Electroactive and antibacterial surgical sutures based on chitosan-gelatin/tannic acid/polypyrrole composite coating. Compos. Part B Eng..

[16] Lou C.-W., Hung C.-Y., Wei M., Li T., Shiu B.-C., Lin J.-H. (2023). Antibacterial Surgical Sutures Developed Using Electrostatic Yarn Wrapping Technology. J. Funct. Biomater..

[17] Zhang Q., Zou Y., Tang L., Liu X., Hu M., Han H., Li Y., Wang F., Wang L., Mao J. (2023). Stage-controlled antibacterial surgical sutures based on curcumin@ ZIF-8 functional coating for improved wound healing. Prog. Org. Coat..

[18] Zhang X., Yang Z., Yang X., Zhang F., Pan Z. (2023). Sustainable Antibacterial Surgical Suture Based on Recycled Silk Resource by an Internal Combination of Inorganic Nanomaterials. ACS Appl. Mater. Interfaces.

[19] Wang X., Liu P., Wu Q., Zheng Z., Xie M., Chen G., Yu J., Wang X., Li G., Kaplan D. (2022). Sustainable antibacterial and anti-inflammatory silk suture with surface modification of combined-therapy drugs for surgical site infection. ACS Appl. Mater. Interfaces.

[20] Altun E., Bayram C., Gultekinoglu M., Matharu R., Delbusso A., Homer-Vanniasinkam S., Edirisinghe M. (2023). Pressure-Spun Fibrous Surgical Sutures for Localized Antibacterial Delivery: Development, Characterization, and In Vitro Evaluation. ACS Appl. Mater. Interfaces.

[21] Syukri D.M., Nwabor O.F., Singh S., Voravuthikunchai S.P. (2021). Antibacterial functionalization of nylon monofilament surgical sutures through in situ deposition of biogenic silver nanoparticles. Surf. Coat. Technol..

[22] Rakhmatullayeva D., Ospanova A., Bekissanova Z., Jumagaziyeva A., Savdenbekova B., Seidulayeva A., Sailau A. (2023). Development and characterization of antibacterial coatings on surgical sutures based on sodium carboxymethyl cellulose/chitosan/chlorhexidine. Int. J. Biol. Macromol..

[23] La Rosa G.R.M., Scapellato S., Cicciù M., Pedullà E. (2024). Antimicrobial Activity of Antibacterial Sutures in Oral Surgery: A Scoping Review. Int. Dent. J..

[24] Wu Q., He C., Wang X., Zhang S., Zhang L., Xie R., Li Y., Wang X., Han Z., Zheng Z. (2021). Sustainable antibacterial surgical suture using a facile scalable silk-fibroin-based berberine loading system. ACS Biomater. Sci. Eng..

[25] Li H., Wang Z., Robledo-Lara J.A., He J., Huang Y., Cheng F. (2021). Antimicrobial surgical sutures: Fabrication and application of infection prevention and wound healing. Fibers Polym..

[26] Al-Sawarees D.K., Darwish R.M., Abu-Zurayk R., Masri M.A. (2024). Assessing silver nanoparticle and antimicrobial combinations for antibacterial activity and biofilm prevention on surgical sutures. J. Appl. Microbiol..

[27] Jamshaid H., Mishra R., Hussain U., Rajput A.W., Tichy M., Muller M. (2022). Natural Fiber Based Antibacterial, Wound Healing Surgical Sutures by the Application of Herbal Antimicrobial Compounds. J. Nat. Fibers.

[28] Chen Y.-G., Li C.-X., Zhang Y., Qi Y.-D., Liu X.-H., Feng J., Zhang X.-Z. (2022). Hybrid suture coating for dual-staged control over antibacterial actions to match well wound healing progression. Mater. Horiz..

[29] Chua R., Lim S.K., Chee C.F., Chin S.P., Kiew L.V., Sim K.S., Tay S.T. (2022). Surgical site infection and development of antimicrobial sutures: A review. Eur. Rev. Med. Pharmacol. Sci..

[30] Matz D., Engelhardt S., Wiencierz A., Soysal S.D., Misteli H., Kirchhoff P., Heizmann O. (2024). Do Antibacterial Skin Sutures Reduce Surgical Site Infections After Elective Open Abdominal Surgery?—A Prospective, Randomized Controlled Single-Center Trial. J. Clin. Med..

[31] Zhang J., Li X., Cheng M., Wan K., Yan S., Peng W., Duan G., Wu Y., Wen L. (2024). MoO3-X nanodots coated suture for combating surgical site infection via antibacterial and anti-inflammatory properties. Nanomed. Nanotechnol. Biol. Med..

[32] Liu M., Zhang Y., Liu K., Zhang G., Mao Y., Chen L., Peng Y., Tao T.H. (2021). Biomimicking antibacterial opto-electro sensing sutures made of regenerated silk proteins. Adv. Mater..

[33] Baygar T., Ugur A., Karaca I.R., Kilinc Y., Gultekin S.E., Sarac N. (2024). Fabrication of a Biocompatible Nanoantimicrobial Suture for Rapid Wound Healing after Surgery. ACS Omega.

[34] Zlobina O., Bugaeva I., Glukhova I., Glukhova A. (2023). Pichkhidze SYa. Experimental modification and investigation of antibacterial surgical suture material. Главный редактoр.

[35] Bhouri N., Debbabi F., Lassoued M.A., Abderrahmen M., Abdessalem S.B. (2022). Wound infections preventing using antibacterial chitosan/Laurus nobilis essential oil emulsion on PET braided surgical sutures. Colloids Surf. A Physicochem. Eng. Asp..

[36] Schmitz N.-D., Ovington L., Berlin J., Zhang S., Collier J. (2023). Optimal usage of antibacterial sutures for wound closure in clinical trials addressing SSI. Lancet.

[37] Mathew S., Kumar K.V., Prabhu A., Shastry R.P., Rajesh K. (2024). Braided silk sutures coated with photoreduced silver nanoparticles for eradicating Staphylococcus aureus and Streptococcus mutans infections. J. Microbiol. Methods.

[38] Basov A., Dzhimak S., Sokolov M., Malyshko V., Moiseev A., Butina E., Elkina A., Baryshev M. (2022). Changes in number and antibacterial activity of silver nanoparticles on the surface of suture materials during cyclic freezing. Nanomaterials.

[39] Schäfer S., Aavani F., Köpf M., Drinic A., Stürmer E.K., Fuest S., Grust A.L.C., Gosau M., Smeets R. (2023). Silk proteins in reconstructive surgery: Do they possess an inherent antibacterial activity? A systematic review. Wound Repair Regen..

[40] Edis Z., Bloukh S.H. (2024). Thymol, a Monoterpenoid within Polymeric Iodophor Formulations and Their Antimicrobial Activities. Int. J. Mol. Sci..

[41] Chen Y.-G., Li C.-X., Zhang Y., Qi Y.-D., Feng J., Zhang X.-Z. (2022). Antibacterial sutures coated with smooth chitosan layer by gradient deposition. Chin. J. Polym. Sci..

[42] Kandathil A.M., Aslam S.A., Abidha R., Cherian M.P., Sudarsanan M. (2023). Evaluation of Microbial Adherence on Antibacterial Suture Materials during Intraoral Wound Healing: A Prospective Comparative Study. J. Contemp. Dent. Pract..

[43] Ranjbar-Mohammadi M., Sa’di V., Moezzi M., Saghafi R. (2022). Fabrication and characterization of antibacterial suture yarns containing PLA/tetracycline hydrochloride-PVA/chitosan nanofibers. Fibers Polym..

[44] Deng X., Qasim M., Ali A. (2021). Engineering and polymeric composition of drug-eluting suture: A review. J. Biomed. Mater. Res. Part A.

[45] Lee Y., Kim H., Kim Y., Noh S., Chun B., Kim J., Park C., Choi M., Park K., Lee J. (2021). A multifunctional electronic suture for continuous strain monitoring and on-demand drug release. Nanoscale.

[46] Deng X., Gould M., Ali M.A. (2021). Fabrication and characterisation of melt-extruded chitosan/keratin/PCL/PEG drug-eluting sutures designed for wound healing. Mater. Sci. Eng. C.

[47] Mendez K., Whyte W., Freedman B.R., Fan Y., Varela C.E., Singh M., Cintron-Cruz J.C., Rothenbücher S.E., Li J., Mooney D.J. (2024). Mechanoresponsive Drug Release from a Flexible, Tissue-Adherent, Hybrid Hydrogel Actuator. Adv. Mater..

[48] Parikh K.S., Omiadze R., Josyula A., Shi R., Anders N.M., He P., Yazdi Y., McDonnell P.J., Ensign L.M., Hanes J. (2021). Ultra-thin, high strength, antibiotic-eluting sutures for prevention of ophthalmic infection. Bioeng. Transl. Med..

[49] Manoukian O.S., Rudraiah S., Arul M.R., Bartley J.M., Baker J.T., Yu X., Kumbar S.G. (2021). Biopolymer-nanotube nerve guidance conduit drug delivery for peripheral nerve regeneration: In vivo structural and functional assessment. Bioact. Mater..

[50] Di X., Liang X., Shen C., Pei Y., Wu B., He Z. (2022). Carbohydrates used in polymeric systems for drug delivery: From structures to applications. Pharmaceutics.

[51] Anup N., Chavan T., Chavan S., Polaka S., Kalyane D., Abed S.N., Venugopala K.N., Kalia K., Tekade R.K. (2021). Reinforced electrospun nanofiber composites for drug delivery applications. J. Biomed. Mater. Res. Part A.

[52] Cazorla-Luna R., Martín-Illana A., Notario-Pérez F., Ruiz-Caro R., Veiga M.-D. (2021). Naturally occurring polyelectrolytes and their use for the development of complex-based mucoadhesive drug delivery systems: An overview. Polymers.

[53] Mu J., Luo D., Li W., Ding Y. (2024). Multiscale polymeric fibers for drug delivery and tissue engineering. Biomed. Technol..

[54] Arun Y., Ghosh R., Domb A.J. (2021). Biodegradable hydrophobic injectable polymers for drug delivery and regenerative medicine. Adv. Funct. Mater..

[55] Osi B., Khoder M., Al-Kinani A.A., Alany R.G. (2022). Pharmaceutical, biomedical and ophthalmic applications of biodegradable polymers (BDPs): Literature and patent review. Pharm. Dev. Technol..

[56] Lee J., Jang E.H., Kim J.H., Park S., Kang Y., Park S., Lee K., Kim J.-H., Youn Y.-N., Ryu W. (2021). Highly flexible and porous silk fibroin microneedle wraps for perivascular drug delivery. J. Control. Release.

[57] Pires P.C., Mascarenhas-Melo F., Pedrosa K., Lopes D., Lopes J., Macário-Soares A., Peixoto D., Giram P.S., Veiga F., Paiva-Santos A.C. (2023). Polymer-based biomaterials for pharmaceutical and biomedical applications: A focus on topical drug administration. Eur. Polym. J..

[58] Rostamitabar M., Abdelgawad A.M., Jockenhoevel S., Ghazanfari S. (2021). Drug-eluting medical textiles: From fiber production and textile fabrication to drug loading and delivery. Macromol. Biosci..

[59] Wu S., Dong T., Li Y., Sun M., Qi Y., Liu J., Kuss M.A., Chen S., Duan B. (2022). State-of-the-art review of advanced electrospun nanofiber yarn-based textiles for biomedical applications. Appl. Mater. Today.

[60] Naser M.A., Sayed A.M., Abdelmoez W., El-Wakad M.T., Abdo M.S. (2024). Biodegradable suture development-based albumin composites for tissue engineering applications. Sci. Rep..

[61] Antoniac I., Antoniac A., Gheorghita D., Gradinaru S. (2021). In vitro study on biodegradation of absorbable suture Materials used for surgical applications. Mater. Plast..

[62] Nouri A., Shirvan A.R., Li Y., Wen C. (2023). Biodegradable metallic suture anchors: A review. Smart Mater. Manuf..

[63] Szabelski J., Karpiński R. (2024). Short-Term Hydrolytic Degradation of Mechanical Properties of Absorbable Surgical Sutures: A Comparative Study. J. Funct. Biomater..

[64] Wu H., Guo T., Zhou F., Bu J., Yang S., Dai Z., Teng C., Ouyang H., Wei W. (2022). Surface coating prolongs the degradation and maintains the mechanical strength of surgical suture in vivo. Colloids Surf. B Biointerfaces.

[65] Li Y., Meng Q., Chen S., Ling P., Kuss M.A., Duan B., Wu S. (2023). Advances, challenges, and prospects for surgical suture materials. Acta Biomater..

[66] Alhulaybi Z.A. (2023). Fabrication and Characterization of Poly (lactic acid)-Based Biopolymer for Surgical Sutures. ChemEngineering.

[67] Mukherjee C., Varghese D., Krishna J., Boominathan T., Rakeshkumar R., Dineshkumar S., Rao C.B., Sivaramakrishna A. (2023). Recent advances in biodegradable polymers–properties, applications and future prospects. Eur. Polym. J..

[68] Al-Shalawi F.D., Hanim M.A., Ariffin M., Kim C.L.S., Brabazon D., Calin R., Al-Osaimi M.O. (2023). Biodegradable synthetic polymer in orthopaedic application: A review. Mater. Today Proc..

[69] Sun S., Chen C., Zhang J., Hu J. (2023). Biodegradable smart materials with self-healing and shape memory function for wound healing. RSC Adv..

[70] Zarinkolah Z., Hosseinkhani S., Nikkhah M. (2021). Investigation on the mechanical, thermal, bio-degradation, and bio-compatibility properties of poly (lactic acid)/poly (ethylene glycol) blend. IIUM Eng. J..

[71] Liu W.-C., Chang C.-H., Chen C.-H., Lu C.-K., Ma C.-H., Huang S.-I., Fan W.-L., Shen H.-H., Tsai P.-I., Yang K.-Y. (2022). 3D-Printed double-helical biodegradable iron suture anchor: A rabbit rotator cuff tear model. Materials.

[72] Chen Y., Sun Y., Wu X., Lou J., Zhang X., Peng Z. (2022). Rotator cuff repair with biodegradable high-purity magnesium suture anchor in sheep model. J. Orthop. Transl..

[73] Yang N., Venezuela J., Zhang J., Wang A., Almathami S., Dargusch M. (2023). Evolution of degradation mechanism and fixation strength of biodegradable Zn–Cu wire as sternum closure suture: An in vitro study. J. Mech. Behav. Biomed. Mater..

[74] Xu L., Liu Y., Zhou W., Yu D. (2022). Electrospun Medical Sutures for Wound Healing: A Review. Polymers.

[75] Akombaetwa N., Bwanga A., Makoni P.A., Witika B.A. (2022). Applications of electrospun drug-eluting nanofibers in wound healing: Current and future perspectives. Polymers.

[76] Han H., Tang L., Li Y., Li Y., Bi M., Wang J., Wang F., Wang L., Mao J. (2023). A multifunctional surgical suture with electroactivity assisted by oligochitosan/gelatin-tannic acid for promoting skin wound healing and controlling scar proliferation. Carbohydr. Polym..

[77] Zhu J., Jin Q., Zhao H., Zhu W., Liu Z., Chen Q. (2021). Reactive oxygen species scavenging sutures for enhanced wound sealing and repair. Small Struct..

[78] Öksüz K.E., Kurt B.m., Şahin İnan Z.D., Hepokur C. (2023). Novel bioactive glass/graphene oxide-coated surgical sutures for soft tissue regeneration. ACS Omega.

[79] Kim K., Kim H., Do S., Kim H. (2023). Potential of Aligned Electrospun PLGA/SIS Blended Nanofibrous Membrane for Tendon Tissue Engineering. Polymers.

[80] Teno J., Pardo-Figuerez M., Evtoski Z., Prieto C., Cabedo L., Lagaron J.M. (2024). Development of Ciprofloxacin-Loaded Electrospun Yarns of Application Interest as Antimicrobial Surgical Suture Materials. Pharmaceutics.

[81] Madheswaran D., Sivan M., Valtera J., Kostakova E.K., Egghe T., Asadian M., Novotny V., Nguyen N.H., Sevcu A., Morent R. (2022). Composite yarns with antibacterial nanofibrous sheaths produced by collectorless alternating-current electrospinning for suture applications. J. Appl. Polym. Sci..

[82] Rivera P., Villegas C., Cabezas R., Pérez B., Torres A., de Dicastillo C.L., Garrido L., Galvez P., Araya C., Romero J. (2023). Development of PLA suture materials by extrusion, electrospinning and supercritical CO_2_ impregnation of ibuprofen and naproxen. J. Supercrit. Fluids.

[83] Mohamadinooripoor R., Kashanian S., Omidfar K. (2023). Poly-ε-Caprolactone/Propolis Electrospun Yarns as Suture. Fibers Polym..

[84] Li Y., Luo H., Li Y., Huang P., Xu J., Zhang J., Cai P., He H., Wu J., Li X. (2023). Surface biofunctional bFGF-loaded electrospun suture accelerates incisional wound healing. Mater. Des..

[85] Yang Z., Liu S., Li J., Wu G., Zhang M., Li F., Jia L., Zhang Y., Li H., Liu X. (2023). Study on Preparation of Core-Spun Yarn Surgical Sutures by Compositing Drug-Loaded Nanofiber Membrane with PLA and Its Controllable Drug Release Performance. Fibers Polym..

[86] Madheswaran D., Sivan M., Hauzerova S., Kostakova E.K., Jencova V., Valtera J., Behalek L., Mullerova J., Nguyen N.H., Capek L. (2024). Continuous fabrication of braided composite nanofibrous surgical yarns using advanced AC electrospinning and braiding technology. Compos. Commun..

[87] Abhari R.E., Snelling S.J., Augustynak E., Davis S., Fischer R., Carr A.J., Mouthuy P.-A. (2024). A hybrid electrospun-extruded polydioxanone suture for tendon tissue regeneration. Tissue Eng. Part A.

[88] Jin D., Yang S., Wu S., Yin M., Kuang H. (2022). A functional PVA aerogel-based membrane obtaining sutureability through modified electrospinning technology and achieving promising anti-adhesion effect after cardiac surgery. Bioact. Mater..

[89] Singh P., Pandey P., Arya D.K., Anjum M.M., Poonguzhali S., Kumar A., Gupta R., Rajamanickam V.M., Singh S., Chaurasia S. (2023). Biomimicking dual drug eluting twisted electrospun nanofiber yarns for post-operative wound healing. Biomed. Mater..

[90] Dang J., Huang S., Li S., Liu J., Chen Z., Wang L., Wang J., Chen H., Xu S. (2024). Effects of the Biomimetic Microstructure in Electrospun Fiber Sutures and Mechanical Tension on Tissue Repair. ACS Appl. Mater. Interfaces.

[91] Pisani S., Croce S., Mauramati S., Marmonti M., Cobianchi L., Herman I., Dorati R., Avanzini M.A., Genta I., Benazzo M. (2022). Engineered Full Thickness Electrospun Scaffold for Esophageal Tissue Regeneration: From In Vitro to In Vivo Approach. Pharmaceutics.

[92] Sharma D., Dhingra S., Banerjee A., Saha S., Bhattacharyya J., Satapathy B.K. (2022). Designing suture-proof cell-attachable copolymer-mediated and curcumin-β-cyclodextrin inclusion complex loaded aliphatic polyester-based electrospun antibacterial constructs. Int. J. Biol. Macromol..

[93] Ye Y., Zhou Y., Jing Z., Xu Y., Yin D. (2021). Electrospun heparin-loaded nano-fiber sutures for the amelioration of achilles tendon rupture regeneration: In vivo evaluation. J. Mater. Chem. B.

[94] Sharma D., Satapathy B.K. (2021). Understanding release kinetics and collapse proof suture retention response of curcumin loaded electrospun mats based on aliphatic polyesters and their blends. J. Mech. Behav. Biomed. Mater..

[95] Nezhentsev A., Abhari R.E., Baldwin M.J., Mimpen J.Y., Augustyniak E., Isaacs M., Mouthuy P.A., Carr A.J., Snelling S.J. (2021). In vitro evaluation of the response of human tendon-derived stromal cells to a novel electrospun suture for tendon repair. Transl. Sports Med..

[96] Li Y., Wang J., Wang Y., Cui W. (2021). Advanced electrospun hydrogel fibers for wound healing. Compos. Part B Eng..

[97] Reddy B., In K.H., Panigrahi B.B., Paturi U.M.R., Cho K., Reddy N. (2021). Modeling tensile strength and suture retention of polycaprolactone electrospun nanofibrous scaffolds by artificial neural networks. Mater. Today Commun..

[98] Li A., Wang L., Qin X. (2024). Manufacturing and Application of Electrospinning Nanofiber Yarn. Electrospinning Fundam. Methods Appl..

[99] Zhang H., Lan D., Wu B., Chen X., Li X., Li Z., Dai F. (2023). Electrospun piezoelectric scaffold with external mechanical stimulation for promoting regeneration of peripheral nerve injury. Biomacromolecules.

[100] Barcena A.J.R., Ravi P., Kundu S., Tappa K. (2024). Emerging Biomedical and Clinical Applications of 3D-Printed Poly(Lactic Acid)-Based Devices and Delivery Systems. Bioengineering.

[101] Wickramasinghe S., Peng C., Ladani R., Tran P. (2022). Analysing fracture properties of bio-inspired 3D printed suture structures. Thin-Walled Struct..

[102] Wickramasinghe S., Do T., Tran P. (2022). Flexural behavior of 3D printed bio-inspired interlocking suture structures. Mater. Sci. Addit. Manuf..

[103] Nash R.J., Li Y. (2021). Experimental and numerical analysis of 3D printed suture joints under shearing load. Eng. Fract. Mech..

[104] Choi D.J., Choi K., Park S.J., Kim Y.-J., Chung S., Kim C.-H. (2021). Suture fiber reinforcement of a 3D printed gelatin scaffold for its potential application in soft tissue engineering. Int. J. Mol. Sci..

[105] Zhou L., Li Y., Tu Q., Wang J. (2023). A 3D printing mold method for rapid fabrication of artificial blood vessels. Colloids Surf. A Physicochem. Eng. Asp..

[106] Xu X., Yu S., Ma L., Mao J., Chen H., Zhu Z., Wang L., Lin H., Zhang J., Wang Z. (2023). Multifunctional high-simulation 3D-printed hydrogel model manufacturing engineering for surgical training. Int. J. Bioprinting.

[107] Altuntas U., Coker D., Yavas D. (2023). Creating tougher interfaces via suture morphology in 3D-printed multi-material polymer composites by fused filament fabrication. Addit. Manuf..

[108] Ye T., Chai M., Wang Z., Shao T., Liu J., Shi X. (2024). 3D-Printed hydrogels with engineered nanocrystalline domains as functional vascular constructs. ACS Nano.

[109] Ghazi A. (2022). A call for change. Can 3D printing replace cadavers for surgical training?. Urol. Clin..

[110] RG A.P., Bajaj G., John A.E., Chandran S., Kumar V.V., Ramakrishna S. (2023). A review on the recent applications of synthetic biopolymers in 3D printing for biomedical applications. J. Mater. Sci. Mater. Med..

[111] Marconi S., Mauri V., Negrello E., Pugliese L., Pietrabissa A., Auricchio F. (2022). Quantitative Assessment of 3D printed blood vessels produced with J750™ digital anatomy™ for suture simulation. bioRxiv.

[112] Wu W., Dong Y., Liu H., Jiang X., Yang L., Luo J., Hu Y., Gou M. (2023). 3D printed elastic hydrogel conduits with 7, 8-dihydroxyflavone release for peripheral nerve repair. Mater. Today Bio.

[113] Laliève L., Adam J., Nataf P., Khonsari R.H. (2021). 3D-printed suture guide for thoracic and cardiovascular surgery produced during the COVID19 pandemic. Ann. 3d Print. Med..

